# Air

**Published:** 1882-01

**Authors:** 


OR THE atmosphere we breathe, is, in its purity, composed of twenty-three parts of oxygen and seventy-seven parts of nitrogen, but it contains particles which do not naturally belong to it. In a damp day the air is full of water or fog, and a pint of it may not contain more than three-fourths of a pint of air ; and as the atmosphere is the thing which acts directly on the blood in the lungs, to withdraw from it all the impurities which it contains, the purer the air is the more capable is it of absorbing the impurities of the blood in the lungs. Hence the purer the air the purer the blood, and the purer the blood the better the health enjoyed in all climes and countries. The purest air is out of doors. There is no pure air within any four walls of a house. You may go into any room, even if it is entirely empty, and a musty or close smell will be immediately observed, much more will there be impurities in the air of our dwellings in proportion to the decaying or odorous things in it—as slops, food, fruits, flowers and the like. That air is best for the health which has no perceptible "smell" about it. The fragrance of the rose and the pink are delicious; but if a person were to sleep in a close room in which there were a great many pinks and roses he would be nearly dead next morning, because the nature of the flowers is such that they are throwing off a multitude of odorous particles every instant, and they being more material, more solid than the air, displace it, so that in every breath there is less air taken into the lungs and more of the substance of the flowers.

A breath of air taken into the lungs may be represented by a piece of fine sponge from which the water has just been squeezed out; put it into a vessel of dirty water; it will take up more of that than if it was half full of water before it was put in. So a breath of pure air taken into the lungs will take up more of the impurities of the blood than it would have done if it had contained or absorbed a large amount of impurities before it went in. Hence the necessity of arranging habitually to breathe the purest air possible. The easiest way to do this is to spend as much of our time in the open air as practicable, and when we are indoors to make it a point to have fresh out-door air coming into our houses all the time; to have fire-place, or door or window, more or less open all the time, day and night, but in such a way as not to come in with a draught.—*Dr. Hall's Health at Home.*

